# The relationship between off-hours admissions for primary percutaneous coronary intervention, door-to-balloon time and mortality for patients with ST-elevation myocardial infarction in England: a registry-based prospective national cohort study

**DOI:** 10.1136/bmjqs-2019-010067

**Published:** 2019-12-12

**Authors:** Sahan Jayawardana, Sebastian Salas-Vega, Felix Cornehl, Harlan M Krumholz, Elias Mossialos

**Affiliations:** 1 Department of Health Policy, London School of Economics and Political Science, London, UK; 2 Center for Outcomes & Evaluation (CORE), Yale University School of Medicine, New Haven, Connecticut, USA; 3 Centre for Health Policy, The Institute of Global Health Innovation, Imperial College London, London, UK

**Keywords:** Healthcare quality improvement, Health policy, Duty Hours/Work hours

## Abstract

**Background:**

The degree to which elevated mortality associated with weekend or night-time hospital admissions reflects poorer quality of care (‘off-hours effect’) is a contentious issue. We examined if off-hours admissions for primary percutaneous coronary intervention (PPCI) were associated with higher adjusted mortality and estimated the extent to which potential differences in door-to-balloon (DTB) times—a key indicator of care quality for ST elevation myocardial infarction (STEMI) patients—could explain this association.

**Methods:**

Nationwide registry-based prospective observational study using Myocardial Ischemia National Audit Project data in England. We examined how off-hours admissions and DTB times were associated with our primary outcome measure, 30-day mortality, using hierarchical logistic regression models that adjusted for STEMI patient risk factors. In-hospital mortality was assessed as a secondary outcome.

**Results:**

From 76 648 records of patients undergoing PPCI between January 2007 and December 2012, we included 42 677 admissions in our analysis. Fifty-six per cent of admissions for PPCI occurred during off-hours. PPCI admissions during off-hours were associated with a higher likelihood of adjusted 30-day mortality (OR 1.13; 95% CI 1.01 to 1.25). The median DTB time was longer for off-hours admissions (45 min; IQR 30–68) than regular hours (38 min; IQR 27–58; p<0.001). After adjusting for DTB time, the difference in adjusted 30-day mortality between regular and off-hours admissions for PPCI was attenuated and no longer statistically significant (OR 1.08; CI 0.97 to 1.20).

**Conclusion:**

Higher adjusted mortality associated with off-hours admissions for PPCI could be partly explained by differences in DTB times. Further investigations to understand the off-hours effect should focus on conditions likely to be sensitive to the rapid availability of services, where timeliness of care is a significant determinant of outcomes.

## Introduction

Internationally, hospital admission during the weekend has been associated with higher mortality rates than during weekdays, a phenomenon that is popularly referred to as the ‘weekend effect’.^1–3^ A ‘night-time effect’ has similarly been observed for night-time admissions.^4^ In the UK, these findings have prompted the National Health Service (NHS) to implement 7-day services based on concerns that the weekend effect reflects inadequate quality of care due to uneven service provision.^5 6^ The nature of some of the measures proposed by policymakers to achieve more consistent 7-day healthcare services has proven controversial. The proposed changes to doctors’ work contracts to increase weekend staffing levels in hospitals were unpopular and resulted in strikes by junior doctors in the NHS.^7^

The issue of the weekend effect has prompted much debate due to questions on the extent to which it exists in the first place, and if so, the range of possible explanations on what causes it.^8 9^ Specifically, the degree to which elevated mortality for patients admitted at weekends and nights reflects poorer quality of care (ie, ‘off-hours effect’) is a contentious issue.^10^ Some have argued that higher mortality for weekend or night-time admissions reflects increased severity of illness, and that in-patient administrative data may not be able to fully account for this.^11 12^

An important consideration is the possibility that an off-hours effect may not exist uniformly across all conditions. In fact, when Bell and Redelmeier demonstrated higher mortality associated with weekend hospital admissions in their seminal study, they posited that a weekend effect would only exist for certain conditions that are more sensitive to the rapid availability of specialised services and personnel.^1^ Therefore, assessing the variation in care quality and outcomes in conditions where there is a high risk of mortality immediately after the onset of clinical events and the timely delivery of care can significantly improve outcomes may provide insights on how care quality is associated with mortality for off-hours admissions.

Current guidelines for the treatment of ST elevation myocardial infarction (STEMI) requires a rapid and coordinated response at time of patient admission.^13^ Any underlying difference in the timely availability of key resources and provision of treatment during off-hours may affect patient outcomes. In the case of STEMI, it remains unclear whether any difference in door-to-balloon (DTB) times—representing the delay between admission and primary percutaneous coronary intervention (PPCI), the preferred reperfusion modality for admitted STEMI patients^13 14^ —accounts for any variation in mortality outcomes based on admission times.

In this study, we made use of data from a nationally mandated population-based acute coronary syndromes (ACS) registry in England to (1) assess if off-hours admissions for PPCI in England were associated with higher adjusted mortality and (2) examine the extent to which potential differences in DTB times between regular and off-hours PPCIs could explain this association.

## Methods

### Data sources and study population

We carried out a registry-based prospective observational cohort study using the Myocardial Ischaemia National Audit Project (MINAP) data, a clinical registry of patients hospitalised with ACS in England and Wales (online supplementary appendix 1 describes the MINAP data collection and validation process). Mandated by the UK Department of Health, MINAP prospectively collects patient-level data from all NHS hospital trusts through standardised data collection forms. Thus, while MINAP may not have as much information on specific procedural characteristics as a local hospital clinical database, the nationally representative nature of the data allows us to assess outcomes at the healthcare system level.

10.1136/bmjqs-2019-010067.supp1 Supplementary data

Linked out-of-hospital mortality data were obtained by MINAP through the UK Office of National Statistics. Identifiable variables were not requested from MINAP; all remaining data were drawn from an established registry and were pseudonymised to the study investigators.

**Figure 1:**
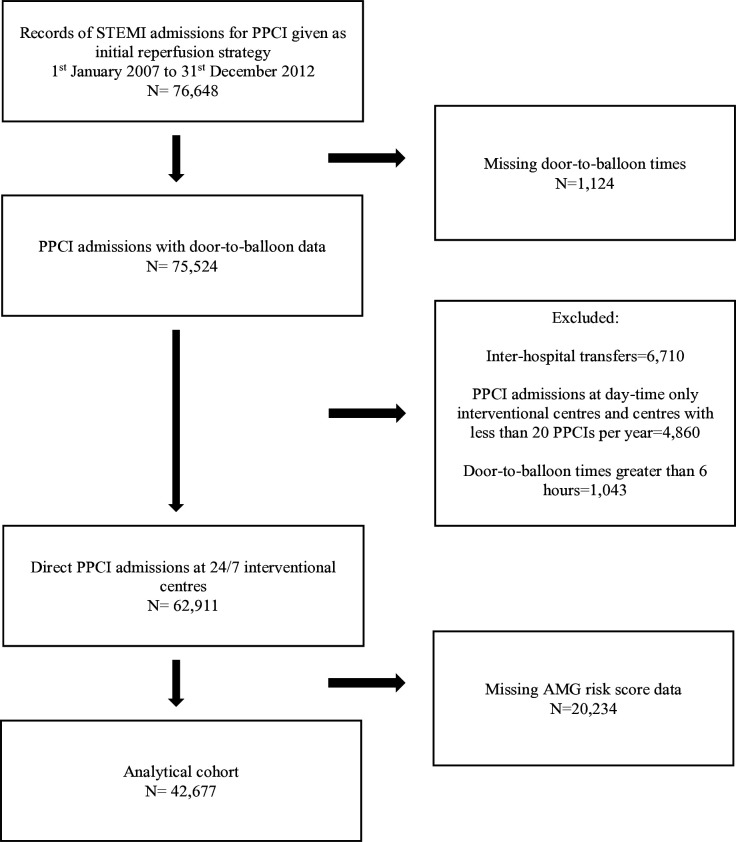
Derivation of analytical cohort. AMG, adjusted mini Global Registry of Acute Coronary Events; PPCI, primary percutaneous coronary intervention; STEMI, ST elevation myocardial infarction.

The analytical cohort for this study consisted of STEMI patients aged over 18 years admitted directly to ‘24/7’ PPCI-capable hospitals for PPCI (figure 1). STEMI patients were identified based on their discharge diagnoses and were selected as having received PPCI according to their initial reperfusion strategy. Hospitals performing only sporadic PPCI procedures, which we defined as less than 20 procedures per year, and only performing PPCIs during regular hours were not included in the analysis. Interhospital transfers were not included in the analysis, and we limited our analysis to PPCIs conducted within 6 hours on hospital arrival on the assumption that patients with a DTB time beyond this did not receive PCI as a primary reperfusion strategy.^15 16^ The analysis was conducted for the time period for which data were available—1 January 2007 to 31 December 2012. We conducted a complete-case analysis (online supplementary tables 1 and 2).

### Study variables

The definitions of the MINAP variables used in the study are listed in online supplementary table 3.

Our primary outcome variable was 30-day mortality. We also examined in-hospital mortality as a secondary outcome. The independent variables were off-hours admissions and DTB time for PPCI. We adopted the NHS definition for out-of-hours periods and defined off-hours admissions as patients admitted for PPCI on weekends and between 18:30 and 7:59 on weekdays. Regular hours were defined as admissions for PPCI between 8:00 and 18:29 on weekdays.^17^ DTB time was measured in minutes and was defined as the interval from the patient’s arrival at the hospital door to the time the first device was used in the coronary artery (balloon, stent or extraction catheter).

We also examined adjusted 30-day and in-hospital mortality outcomes for PPCIs using an alternative definition of off-hours as patients admitted on weekends and between 19:00 and 6:59 on weekdays. Additionally, the above-mentioned mortality outcomes were analysed for the analytical cohort including inter-hospital transfers, day-time only centres and PCI cases with DTB times greater than 6 hours.

### Statistical analysis

We described patient characteristics using percentages for categorical data, means and SD or medians and IQRs for normally and non-normally distributed continuous variables, respectively. Statistical comparisons for differences in baseline characteristics among patients admitted during regular hours and off-hours were performed using χ^2^ tests for categorical variables, t-tests and Wilcoxon rank sum tests for normally and non-normally distributed continuous variables, respectively. DTB times were described using median and IQR based on time of admission. All p values were calculated as two-tailed analyses, using a significance level of 5%.

To determine whether off-hours PPCI admissions were associated with a higher likelihood of adjusted 30-day and in-hospital mortality, we fitted hierarchical logistic regression models.^18^ A two-level modelling strategy was adopted to allow for the clustering of patients within hospitals by including the hospitals as random effects with a variance components covariance structure. The models were adjusted for baseline patient characteristics and risk factors that are relevant to STEMI including gender, cardiovascular disease history (previous acute myocardial infarction, angina, peripheral vascular disease, cerebrovascular disease (stroke), percutaneous coronary intervention, coronary artery bypass grafting and chronic renal failure) and cardiovascular risk factors (diabetes, smoking status, hypercholesterolemia, hypertension, asthma/chronic obstructive pulmonary disease and family history of coronary heart disease).^15 19^

We used the adjusted mini-GRACE (AMG) risk score to adjust for patient case-mix. The AMG risk score was derived from the Global Registry of Acute Coronary Events (GRACE) risk score, modified and adopted for MINAP data by the National Institute for Health and Care Excellence.^20 21^ The AMG risk score was calculated using eight variables from MINAP: age, heart rate, admission systolic blood pressure, electrocardiographic ST segment deviation, cardiac arrest, elevated cardiac markers (cardiac troponin concentration >0.05 ng/mL), use of a loop diuretic and creatinine concentration. The AMG risk score has demonstrated good performance in terms of discriminative ability and predictive accuracy for 6-month mortality in patients hospitalised for STEMI and has been validated as appropriate to use for the retrospective adjustment of patient case-mix in MINAP data.^22^

We included hospital annual PPCI volume to account for potential hospital volume effects on outcomes. To account for seasonality effects and aggregate time trends, we included controls for month and year. We also controlled for deprivation using the Index of Multiple Deprivation (included as deciles), which ranks small areas in England (Lower-layer Super Output areas) from one (most deprived area) to 32 844 (least deprived area).

Patient DTB times were subsequently added to our logistic regression model (as a continuous variable) to determine if accounting for DTB times changed our previous adjusted point estimates for patient mortality following PPCIs performed during off-hours relative to regular hours. A significant change in the point estimates would be indicative of DTB times being a mediating factor of any association that may exist between admission time for PPCI and mortality.

Overall model performance was assessed using the area under the receiver operating characteristics curve (C statistic).

Statistical analyses was performed using STATA MP V.14.

## Results

### Patient characteristics

Our analytical cohort consisted of 42 677 patients directly admitted for PPCI to PPCI-capable hospitals in England between 2007 and 2012. Overall, 56% of these admissions occurred during off-hours. Table 1 presents the baseline characteristics of the patients admitted for PPCI during regular hours and off-hours.

**Table 1:** Baseline patient characteristics

	Regular hours (n=18 452)	Off-hours (n=24 225)	P value
Age (SD), years	64.52 (12.95)	62.86 (12.92)	<0.001
Gender—female (n (%))	4801 (26.02)	6093 (25.15)	0.042
Deprivation, most deprived decile (n (%))	2623 (14.22)	3498 (14.44)	0.536
**Medical history**			
Previous AMI (n (%))	2187 (11.85)	2927 (12.08)	0.468
Previous angina (n (%))	2170 (11.76)	2815 (11.62)	0.655
History of hypertension (n (%))	7503 (40.66)	9598 (39.62)	0.030
History/present PVD (n (%))	465 (2.52)	552 (2.28)	0.105
History of stroke/CVD (n (%))	809 (4.38)	921 (3.80)	0.003
History of asthma or COPD (n (%))	1867 (10.12)	2414 (9.96)	0.602
History of chronic renal failure (n (%))	377 (2.04)	346 (1.43)	<0.001
History/present hypercholesterolemia (n (%))	5733 (31.07)	7450 (30.75)	0.483
Previous PCI (n (%))	1522 (8.25)	1878 (7.75)	0.061
Previous CABG (n (%))	343 (1.86)	494 (2.04)	0.183
Family history of premature CHD (n (%))	6169 (33.43)	8106 (33.46)	0.951
Current smoker (n (%))	6725 (36.45)	10 050 (41.49)	<0.001
Diabetes (n (%))	2290 (12.41)	3018 (12.46)	0.883
**Clinical presentation**			
Heart rate b.p.m (IQR)	75 (64–87)	75 (64–88)	0.016
Systolic BP mmHg (SD)	131.98 (27.49)	132.88 (27.82)	0.001
Elevated cardiac markers (n (%))	17 016 (92.22)	22 097 (91.22)	<0.001
Cardiac arrest (n (%))	1804 (9.78)	2459 (10.15)	0.202
Creatinine umol/L(IQR)	85 (72–100)	85 (73–101)	0.015
Loop diuretic (n (%))	2428 (13.16)	3424 (14.13)	0.004
Adjusted mini-GRACE score (SD)	118.43 (30.63)	115.29 (30.71)	<0.001

AMI, acute myocardial infarction; BP, blood pressure; CABG, coronary artery bypass surgery; CHD, coronary heart disease; COPD, chronic obstructive pulmonary disease; CVD, cerebrovascular disease; GRACE, Global Registry of Acute Coronary Events; PCI, percutaneous coronary intervention; PVD, peripheral vascular disease.

The regular hours cohort had a lower proportion of current smokers but were on average, older, and had a larger proportion of patients with a history of stroke and chronic renal failure. They were also generally sicker based on clinical risk factors, as reflected by the higher average AMG risk score compared with the off-hours group (mean AMG score 118.4 vs 115.3, p<0.001). There were no statistically significant differences between the two groups with respect to the other baseline patient characteristics included in the study.

### Association between adjusted mortality and off-hours PPCI admissions

Overall, there was no statistically significant difference in unadjusted 30-day or in-hospital mortality rate between the two groups. The unadjusted 30-day mortality rate was 3.91% for PPCI admissions during regular hours and 3.86% for admissions during off-hours (p=0.80). The unadjusted in-hospital mortality rates for regular and off-hours admissions were 2.81% and 2.84% (p=0.82), respectively.

After adjusting for the patient risk factors described in table 1 and controlling for hospital PPCI volume, seasonality and time trend effects, patients admitted for PPCI during off-hours had a higher likelihood of 30-day mortality (OR 1.13; 95% CI 1.01 to 1.25; p=0.02) and in-hospital mortality (OR 1.16; 95% CI 1.02 to 1.32; p=0.02) (table 2). Online supplementary tables 4 and 5 present full model results.

**Table 2:** Adjusted 30-day and in-hospital mortality by time of admission for PPCI

	Adjusted OR^a^ for off-hours (95% CI)*	Adjusted OR^b^ for off-hours (95% CI)†
30-day mortality	1.13 (1.01 to 1.25; p=0.02)	1.08 (0.97 to 1.20; p=0.15)
In-hospital mortality	1.16 (1.02 to 1.32; p=0.02)	1.09 (0.95 to 1.24; p=0.18)

*Adjusted OR^a^—obtained using a hierarchical logistic regression model that adjusted for AMG risk score, sex, Index of Multiple Deprivation score, previous acute myocardial infarction, angina, peripheral vascular disease, cerebrovascular disease (stroke), percutaneous coronary intervention, coronary artery bypass grafting, chronic renal failure, diabetes, smoking status, hypercholesterolemia, hypertension, asthma/chronic obstructive pulmonary disease, family history of coronary heart disease, annual hospital PPCI volume and month and year of admission. Hospitals included as random intercepts (46 ‘24/7’ interventional centres included).

†Adjusted OR^b^—all variables from OR^a^ plus DTB time.

AMG, adjusted mini-Global Registry of Acute Coronary Events; DTB, door-to-balloon; p, p-value; PPCI, primary percutaneous coronary intervention.

The model had good discriminative value, strongly predictive of mortality, with a C-statistic of 0.86 for 30-day mortality and 0.91 for in-hospital mortality.

### DTB times

The median DTB time for PPCI was longer during off-hours (45 min; IQR 30–68) than regular hours (38 min; IQR 27–58), a statistically significant difference of 7 min (p<0.001). Weekday night-time admissions for PPCI had higher median DTB times than the corresponding weekday daytime admissions (table 3). During the weekend, both daytime and night-time admissions for PPCI had median DTB times that were not significantly different to weekday night-time admissions (p=0.25).

**Table 3:** Median DTB times for PPCI by day and time of admission

	Median DTB time (25th–75th percentile), min						
Day of admission	Monday	Tuesday	Wednesday	Thursday	Friday	Saturday	Sunday
Regular hours	40 (28–60)	38 (27–56)	38 (27–57)	38 (27–59)	38 (27–58)	45 (30–70)	45 (30–69)
Off-hours	44 (30–65)	45 (30–69)	45 (30–69)	45 (30–67)	44 (29–68)	45 (30–69)	44 (30–66)

DTB, door-to-balloon; PPCI, primary percutaneous coronary intervention.

Overall, there was a modest increase in the gap in DTB times between regular and off-hours PPCIs at higher percentiles—for example, at the 95th percentile, the DTB times were 118 and 131 min, respectively (online supplementary figure 1).

### DTB times and the off-hours effect

When we accounted for PPCI DTB time in the hierarchical logistic regression models, the difference in adjusted 30-day mortality and in-hospital mortality between regular and off-hours PPCI admissions was attenuated and no longer statistically significant (table 2). Online supplementary tables 6 and 7 present full model results.

Our results were robust to using an alternative definition of off-hours—patients admitted for PPCI during off-hours had a significantly higher likelihood of adjusted 30-day and in-hospital mortality compared with regular hours, and after controlling for DTB time, the difference in adjusted 30-day and in-hospital mortality was attenuated and no longer statistically significant (online supplementary table 8). In addition, when the analyses were conducted for the analytical cohort including inter-hospital transfers, day-time only centres and PCI cases with DTB times greater than 6 hours, our results did not significantly differ (online supplementary table 9).

The relationship between DTB times and mortality is detailed in online supplementary appendix 2.

## Discussion

In this study, we assessed if off-hours admissions of STEMI patients undergoing PPCIs in England were associated with higher adjusted mortality and evaluated the extent to which potential differences in DTB times between regular and off-hours PPCIs could explain this association. First, we found admissions for PPCI during off-hours were significantly associated with higher adjusted 30-day and in-hospital mortality compared with admissions during regular hours. Second, we confirmed small but significant differences in DTB times between regular and off-hours PPCIs—DTB times were, on average, longer for patients admitted during off-hours. After accounting for DTB time, the difference in adjusted 30-day and in-hospital mortality between regular and off-hours PPCIs was attenuated and no longer statistically significant, suggesting that higher adjusted mortality associated with off-hours admissions could be partly explained by differences in DTB times.

While previous studies have found elevated mortality associated with weekend or night-time admissions, the reason why this phenomenon generates debate is the implication of poorer quality of care during off-hours admissions. If, for instance, elevated mortality during off-hours entirely reflected higher severity of illness, this would not necessarily be an ‘off-hours effect’ in the sense that the term usually generates debate. Potential selection bias may explain some of the excess mortality associated with off-hours admissions, where routinely collected in-patient administrative data may not be able to fully account for severity of illness.^12 23^ Some have made this argument based on the observed lower volume of patients admitted from the community and accident and emergency department (A&E) at weekends, as well as the observed higher proportions of patients admitted during off-hours arriving by ambulance (proxy for severity of illness), among a patient population admitted in an emergency after attending A&E.^11 24^

Previous studies that assessed STEMI mortality outcomes based on admission times found discrepant findings but differed widely on study design. Some were limited to a single high-volume PCI centre,^25 26^ some did not account for DTB times,^27^ others assessed a relatively small sample of patients within a single region,^28 29^ and some relied on a voluntary database, making it difficult to generalise the evidence to a wider population.^30–32^

We made use of a national clinical registry of ACS patients in England to better adjust for illness severity by accounting for clinical risk factors specific to STEMI. We focused on PPCI, which is superior to fibrinolytic therapy if performed rapidly by a team of experts^33^ but its effectiveness could be limited by delays in delivery.^34^ DTB time is a key indicator of quality of care for STEMI patients treated with PPCI,^35^ and delays in DTB time have been consistently associated with poorer outcomes.^36 37^ A time-sensitive indicator of care quality such as DTB time is more likely to be sensitive to potential variations in the availability of key resources, personnel and decision makers than other quality indicators of STEMI care. We were able to use information on DTB times for PPCIs performed in England to investigate how DTB time varied by day and time of admission and assess the extent to which these differences explained the higher adjusted mortality for off-hours PPCIs.

Our findings should be interpreted with several limitations in mind. First, even though we have taken measures to carefully adjust for patient risk factors, potential biases may have been introduced by unobserved patient factors that might vary based on time of presentation. In addition, we had to exclude patients with missing AMG risk score data. Fifty-four per cent of admissions in this excluded group were during off-hours, similar to the proportion of off-hours admissions in our analytical cohort (56%). The DTB times and unadjusted mortality rates for this excluded group are reported in online supplementary tables 1 and 2. The median DTB times for PPCIs were longer in this group but the difference in median DTB time between regular and off-hours admissions was in the same direction as the analytical cohort in our study (44 min during regular hours and 54 min in off-hours). The unadjusted 30-day and in-hospital mortality rates were also higher for the excluded group but this was the case for both regular and off-hours PPCIs. Nonetheless, we cannot rule out potential bias introduced to our analysis by omitting this group due to missing AMG risk score data, which may affect the generalisability of our results to the whole population of STEMI patients receiving PPCI.

Second, we lacked information on hospital-level factors—we did not have access to the name or location of the hospitals. Further, MINAP does not have information on hospital staffing levels. Therefore, we could not assess how these factors may contribute to variations in DTB times and PPCI outcomes. Third, we did not have access to potentially important clinical variables on procedural characteristics (eg, patients with complex lesions, bifurcation lesions etc).

In assessing the reperfusion times for STEMI patients in England, it should be noted that the DTB times reported in this study show good overall performance, both in terms of the proportion of PPCIs conducted within the 90 min benchmark recommended by clinical practice guidelines^13^ (90.27% of PPCIs during regular hours and 86.08% during off-hours in the analytical cohort), and also compared with the reperfusion times achieved in other countries. For example, in Sweden, a median 74% of STEMI patients received reperfusion treatment within the recommended time in 2013.^38^ In USA, in 2011, the median DTB time for PPCI was estimated to be 63 min (IQR 47–80).^19^

Bell and Redelmeier considered acute myocardial infarction (AMI) a ‘control’ condition, where a weekend effect was not anticipated and not found in their study.^1^ However, this was during a period (1988 to 1997) when AMI patients were given thrombolytic treatment, where there would be less reasons to expect an off-hours effect.^39 40^ Current guideline indicated care for STEMI patients contain some of the criteria posited by Bell and Redelmeier for conditions where a weekend effect would be anticipated. By accounting for DTB time, an important quality measure in the care pathway for STEMI patients, we have shown that higher mortality associated with off-hours PPCIs is likely to be explained, in part, by longer DTB times during off-hours. This suggests that conditions requiring a rapid and coordinated response, where timeliness of care is an important determinant of outcomes, are more likely to be affected by the less timely availability of key resources and personnel, showing reduced performance on some quality measures during off-hours. Importantly, this also highlights the specific circumstances in which variations in care quality could explain the off-hours effect. It is likely that for many other conditions, higher mortality associated with off-hours admissions largely reflects higher severity of illness.

The logical next step would be to understand the stages in the diagnostic, decision-making and treatment pathways of STEMI patients where potential delays occur that may lead to longer DTB times for off-hours admissions. Policy measures to address the off-hours effect should be based on a detailed understanding of how and why care quality varies in the care pathway for acute conditions that are more sensitive to the rapid availability of key resources and personnel. Therefore, it would be beneficial for future research to focus on understanding the temporal variation in care quality for these conditions, where the timely delivery of care can significantly improve outcomes—for example, Bray and colleagues found several patterns of variation in different aspects of quality in acute stroke care.^41^ A better understanding of the underlying factors driving temporal variation in care quality would enable policymakers to appropriately target resources towards conditions where disparities in care quality during off-hours could be reduced in a cost-effective manner.
