# Supplementary material for: The relationship between off-hours admissions for primary percutaneous coronary intervention, door-to-balloon time and mortality for patients with ST-elevation myocardial infarction in England: a registry-based prospective national cohort study
Source: BMJ Qual Saf. 2019 Dec 12;29(7):541–9. doi: 10.1136/bmjqs-2019-010067 (PMC7362773; doi:10.1136/bmjqs-2019-010067)
Supplement: Supplementary data [file bmjqs-2019-010067supp001.pdf]

The relationship between off-hours admissions for primary percutaneous coronary intervention, door-to-balloon time and mortality for patients with ST-elevation myocardial infarction in England: a registry-based prospective national cohort study

Supplementary material

Contents

Appendix 1: The Myocardial Ischaemia National Audit Project (MINAP) registry – data collection and data quality 1

Supplementary Table 1: Door-to-balloon times for excluded cases due to missing adjusted mini-GRACE (AMG) risk score variables 2

Supplementary Table 2: Mortality outcomes for excluded cases due to missing adjusted mini-GRACE (AMG) risk score variables 2

Supplementary Table 3: MINAP variable definitions 3

Supplementary Table 4 – Hierarchical logistic regression model, 30-day mortality without adjustment for DTB times 5

Supplementary Table 5 – Hierarchical logistic regression model, in-hospital mortality without adjustment for DTB times 7

Supplementary Figure 1: Distribution of door-to-balloon times for PPCI patients – 2007-2012 9

Supplementary Table 6 – Hierarchical logistic regression model, 30-day mortality with adjustment for DTB times 10

Supplementary Table 7 – Hierarchical logistic regression model, in-hospital mortality with adjustment for DTB times 12

Supplementary Table 8 – Adjusted 30-day and in-hospital mortality by time of admission for PPCI (alternative definition of off-hours [1]) 14

Supplementary Table 9 - Adjusted 30-day and in-hospital mortality by time of admission [1] for PPCI (for analytical cohort including inter-hospital transfers, day-time only centres and PCI cases with DTB times greater than 6 hours) 15

Appendix 2: Door-to-balloon times and mortality 16

References 18

Appendix 1: The Myocardial Ischaemia National Audit Project (MINAP) registry – data collection and data quality

The Myocardial Ischaemia National Audit Project (MINAP) accrues data for episodes of care from patients with acute coronary syndromes (ACS) admitted to all acute National Health Service (NHS) hospitals in England and Wales. [1] Hospitals use MINAP software (secure web portal), locally developed software or commercial applications to enter data which are then uploaded to central servers managed by the National Institute for Cardiovascular Outcomes Research (NICOR). Data is captured via manual entry by clinical audit staff, nurses or doctors. [2] The costs of local data entry are borne by the participating hospitals but non-financial incentives operate at the hospital level. [1]

To ensure data quality, annual validation exercises are conducted, where every hospital is required to re-enter 20 key fields from the case notes of 20 randomly selected patients. Agreement between the original and re-entered data is assessed and sent to hospitals to help them identify areas of improvement. [2] The median level of agreement between MINAP data and re-audit data was 89.5% in 2008. [1]

Jayawardana S, *et al. BMJ Qual Saf* 2020; 29:541–549. doi: 10.1136/bmjqs-2019-010067

Supplementary Table 1: Door-to-balloon times for excluded cases due to missing adjusted mini-GRACE (AMG) risk score variables

	Regular hours(N=9,217)	Off-hours(N=11,017)
Door-to-balloon times(IQR)	44(32-65)	54(37-79)

Supplementary Table 2: Mortality outcomes for excluded cases due to missing adjusted mini-GRACE (AMG) risk score variables

	Regular hours(N=7,974)	Off-hours(N=9,761)
30-day mortality%	7.54%	6.72%
In-hospital mortality%	6.36%	5.50%

Jayawardana S, *et al. BMJ Qual Saf* 2020; 29:541–549. doi: 10.1136/bmjqs-2019-010067

Supplementary Table 3: MINAP variable definitions

Variable	Description
Time of arrival at hospital	Time of arrival of the ambulance at the front door (time recorded by ambulance service).
Time of reperfusion treatment	Time of onset of lytic treatment (infusion or injection). The time the first device is used in coronary artery (balloon, stent or extraction catheter). It is not the time the angioplasty guidewire is first introduced, even if this restores flow.
Initial reperfusion treatment: pPCI	Primary PCI for STEMI/LBBB
Discharge diagnosis - Myocardial infarction (ST elevation)	There should be a history consistent with the diagnosis. The diagnosis requires the presence of (new) cardiographic changes of ST elevation consistent with infarction of =>2mm in contiguous chest leads and/or ST elevation of =>1 mm ST elevation in 2 or more standard leads. (New LBBB is included; although new ST elevation may be apparent in the presence of LBBB). There must be troponin elevation above the local reference range This group includes all patients with STEMI regardless of whether typical changes were evident on the initial ECG or developed subsequently. If ST elevation is present on any ECG during the episode in association with elevated troponin, then the diagnosis must be Myocardial infarction (ST elevation).
Heart rate	The heart rate is recorded from the first ECG after admission to hospital, whilst in a stable cardiac rhythm i.e. sinus rhythm, or chronic AF. In complete heart block, ventricular rate recorded.
Systolic BP	The first systolic blood pressure recorded after index admission to Hospital.
Elevated cardiac markers	Identifies if cardiac biomarkers are raised during the acute phase of admission.
Cardiac arrest	Cardiac arrests for patients with infarction who arrest in hospital. Also includes patients with infarction who have an out of hospital cardiac arrest and who survive to be admitted to hospital.
Creatinine	Recorded within 24 hours of index admission (micromol/L.) There is an in-built range check checking that the entered value is 30 – 1000 micromol/L.
Loop diuretic	Given at admission and drug continued or introduced while in hospital.
Previous AMI	Any previously validated episode of acute myocardial infarction.
Previous angina	Symptoms due to cardiac ischaemia developing or already in existence at least 2 weeks prior to admission, and continuing up to admission.
History of hypertension	A patient already receiving treatment (drug, dietary or lifestyle) for hypertension or with recorded BP >140/90 on at least 2 occasions before admission.
History / present PVD	Presence of peripheral vascular disease, either presently symptomatic or previously treated. Include renovascular disease and aortic aneurysm.
History of stroke / CVD	A history of cerebrovascular ischaemia, including transient cerebral ischaemic episodes as well as events with deficit lasting >24 hours.
History of asthma or COPD	Any form of obstructive airways disease.
History of chronic renal failure	Defined as creatinine consistently more than 200 micromol/L.
History / present hypercholesterolemia	A value recorded during the first 24 hours after index admission. There is an in-built range check checking that the entered value is 2.5 – 25 mmol/L.
Previous PCI	A percutaneous coronary intervention at any time prior to this admission.
Previous CABG	Coronary artery bypass grafting at any time prior to this admission.

Jayawardana S, *et al. BMJ Qual Saf* 2020; 29:541–549. doi: 10.1136/bmjqs-2019-010067

Family history of premature CHD	Identifies a family history of premature CHD by diagnosis in males before 55 years or females before 65 years.

Source: NICOR: Data collection for the Myocardial Ischaemia National Audit Project – Application Notes

Jayawardana S, *et al. BMJ Qual Saf* 2020; 29:541–549. doi: 10.1136/bmjqs-2019-010067

Supplementary Table 4 – Hierarchical logistic regression model, 30-day mortality without adjustment for DTB times

Variable (reference)	30-day mortality Odds-ratio	95% confidence interval	Wald p-value
-Intercept-	-	-	<0.001
Off-hours*	1.13	(1.01, 1.25)	0.02
AMG score	1.04	(1.04, 1.05)	<0.001
Female patient	0.91	(0.81, 1.02)	0.12
Deprivation (most deprived)	1.00	-	
2	0.82	(0.65, 1.03)	0.09
3	0.89	(0.71, 1.11)	0.32
4	0.88	(0.70, 1.11)	0.30
5	0.80	(0.64, 1.01)	0.07
6	0.90	(0.72, 1.13)	0.40
7	0.75	(0.59, 0.95)	0.01
8	0.73	(0.58, 0.93)	0.01
9	0.66	(0.52, 0.84)	<0.01
10	0.65	(0.50, 0.84)	<0.01
Previous AMI	0.96	(0.80, 1.15)	0.67
Previous angina	0.97	(0.82, 1.15)	0.77
History of hypertension	0.87	(0.77, 0.97)	0.02
History/present PVD	1.11	(0.84, 1.46)	0.45
History of stroke/CVD	1.19	(0.97, 1.45)	0.07
History of asthma/COPD	1.04	(0.88, 1.22)	0.62
History of CRF	1.36	(1.04, 1.78)	0.02
History/present hypercholesterolemia	0.79	(0.69, 0.90)	<0.01
Previous PCI	0.90	(0.71, 1.14)	0.41
Previous CABG	0.75	(0.52, 1.10)	0.14
Family history of premature CHD	0.77	(0.67, 0.88)	<0.001
Current smoker	0.98	(0.86, 1.13)	0.88
Diabetes	1.71	(1.46, 1.97)	<0.001
Month (January)	1.00	-	
February	1.01	(0.77, 1.33)	0.90
March	1.16	(0.89, 1.52)	0.24
April	1.20	(0.93, 1.56)	0.15
May	0.94	(0.72, 1.23)	0.68
June	1.07	(0.82, 1.40)	0.59
July	0.94	(0.72, 1.23)	0.69
August	1.03	(0.76, 1.35)	0.77
September	0.94	(0.72, 1.23)	0.67
October	1.02	(0.78, 1.32)	0.86

Jayawardana S, *et al. BMJ Qual Saf* 2020; 29:541–549. doi: 10.1136/bmjqs-2019-010067

November	1.07	(0.82, 1.38)	0.59
December	1.05	(0.81, 1.36)	0.68
Year (2007)	1.00	-	
2008	0.92	(0.66, 1.27)	0.62
2009	1.09	(0.80, 1.48)	0.57
2010	1.09	(0.80, 1.49)	0.55
2011	0.95	(0.70, 1.30)	0.79
2012	1.04	(0.77, 1.42)	0.75
Annual hospital PPCI volume	0.99	(0.99, 0.99)	<0.001
Annual hospital PPCI volume squared	1.00	(1.00, 1.00)	<0.001
**Random-effect standard deviation estimate**	Hospital = 0.24		

* Average marginal effects dy/dx is 0.122

Supplementary Table 5 – Hierarchical logistic regression model, in-hospital mortality without adjustment for DTB times

Variable (reference)	In-hospital mortality Odds-ratio	95% confidence interval	Wald p-value
-Intercept-	-	-	<0.001
Off-hours*	1.16	(1.02, 1.32)	0.02
AMG score	1.05	(1.05, 1.06)	<0.001
Female patient	0.94	(0.82, 1.07)	0.38
Deprivation (most deprived)	1.00	-	
2	0.79	(0.60, 1.04)	0.09
3	0.74	(0.56, 0.98)	0.04
4	0.89	(0.68, 1.18)	0.45
5	0.78	(0.59, 1.03)	0.08
6	0.85	(0.65, 1.12)	0.26
7	0.64	(0.48, 0.86)	<0.01
8	0.68	(0.50, 0.90)	<0.01
9	0.72	(0.54, 0.96)	0.02
10	0.62	(0.45, 0.85)	<0.01
Previous AMI	0.94	(0.76, 1.16)	0.58
Previous angina	1.00	(0.83, 1.21)	0.96
History of hypertension	0.85	(0.74, 0.97)	0.02
History/present PVD	1.22	(0.89, 1.67)	0.21
History of stroke/CVD	1.09	(0.86, 1.37)	0.46
History of asthma/COPD	1.02	(0.85, 1.24)	0.77
History of CRF	1.26	(0.92, 1.72)	0.14
History/present hypercholesterolemia	0.77	(0.66, 0.90)	<0.001
Previous PCI	0.97	(0.74, 1.28)	0.87
Previous CABG	0.80	(0.52, 1.24)	0.33
Family history of premature CHD	0.73	(0.61, 0.86)	<0.001
Current smoker	1.15	(0.98, 1.35)	0.08
Diabetes	1.67	(1.42 1.97)	<0.001
Month (January)	1.00	-	
February	0.91	(0.65, 1.27)	0.58
March	1.18	(0.86, 1.61)	0.29
April	1.16	(0.85, 1.58)	0.34
May	0.92	(0.67, 1.27)	0.63
June	1.04	(0.75, 1.42)	0.80
July	0.86	(0.62, 1.19)	0.37
August	1.10	(0.80, 1.51)	0.53
September	0.96	(0.70, 1.32)	0.82
October	0.95	(0.70, 1.30)	0.76

Jayawardana S, *et al. BMJ Qual Saf* 2020; 29:541–549. doi: 10.1136/bmjqs-2019-010067

November	0.97	(0.71, 1.33)	0.89
December	0.99	(0.73, 1.34)	0.96
Year (2007)	1.00	-	
2008	0.86	(0.59, 1.25)	0.44
2009	0.92	(0.64, 1.33)	0.68
2010	0.99	(0.68, 1.45)	0.99
2011	0.78	(0.53, 1.14)	0.20
2012	1.00	(0.68, 1.46)	0.97
Annual hospital PPCI volume	0.99	(0.99, 0.99)	<0.001
Annual hospital PPCI volume squared	1.00	(1.00, 1.00)	<0.01
**Random-effect standard deviation estimate**	Hospital = 0.44		

* Average marginal effects dy/dx is 0.154

Supplementary Figure 1: Distribution of door-to-balloon times for PPCI patients – 2007-2012

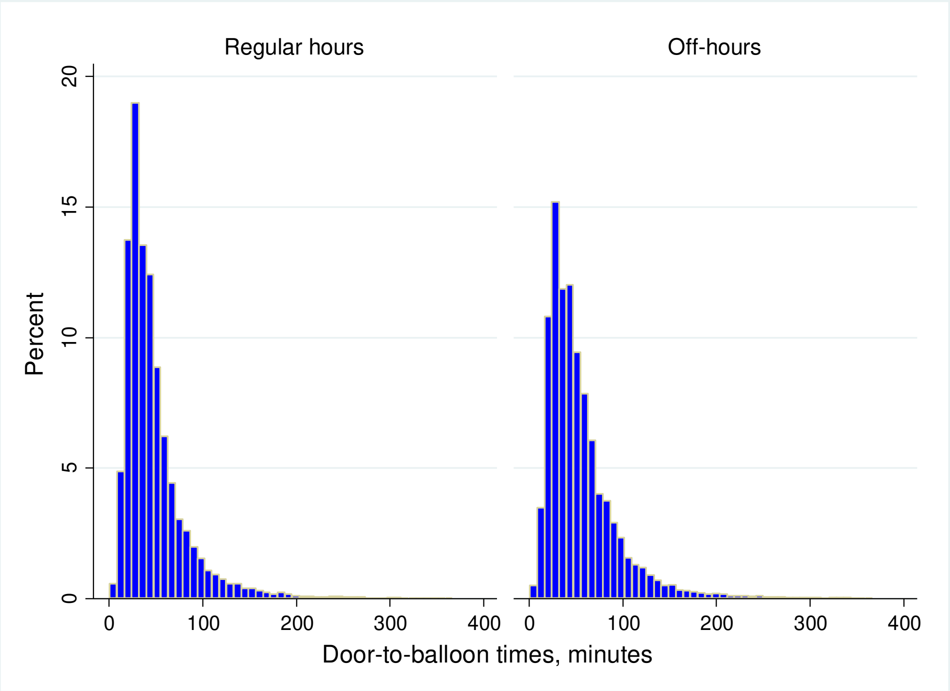

Percentiles – DTB times (minutes)		
	Regular hours	Off-hours
1%	10	11
**5%**	16	18
**10%**	20	22
**25%**	27	30
**50%**	38	45
**75%**	58	68
**90%**	89	101
**95%**	118	131
**99%**	209	227

Jayawardana S, *et al. BMJ Qual Saf* 2020; 29:541–549. doi: 10.1136/bmjqs-2019-010067

Supplementary Table 6 – Hierarchical logistic regression model, 30-day mortality with adjustment for DTB times

Variable (reference)	30-day mortality Odds-ratio	95% confidence interval	Wald p-value
-Intercept-	-	-	<0.001
Off-hours	1.08	(0.97, 1.20)	0.15
AMG score	1.04	(1.04, 1.05)	<0.001
DTB time (per 10 min increase)	1.04	(1.03, 1.05)	<0.001
Female patient	0.90	(0.81, 1.02)	0.10
Deprivation (most deprived)	1.00	-	
2	0.81	(0.65, 1.02)	0.08
3	0.90	(0.72, 1.13)	0.38
4	0.89	(0.71, 1.13)	0.36
5	0.81	(0.65, 1.03)	0.09
6	0.92	(0.73, 1.16)	0.50
7	0.76	(0.60, 0.97)	0.03
8	0.76	(0.60, 0.96)	0.02
9	0.68	(0.53, 0.86)	<0.01
10	0.66	(0.51, 0.86)	<0.01
Previous AMI	0.94	(0.79, 1.14)	0.58
Previous angina	0.96	(0.81, 1.13)	0.63
History of hypertension	0.86	(0.77, 0.97)	0.01
History/present PVD	1.09	(0.82, 1.44)	0.54
History of stroke/CVD	1.18	(0.96, 1.44)	0.09
History of asthma/COPD	1.02	(0.86, 1.20)	0.79
History of CRF	1.34	(1.02, 1.75)	0.03
History/present hypercholesterolemia	0.79	(0.69, 0.90)	<0.001
Previous PCI	0.91	(0.72, 1.15)	0.44
Previous CABG	0.70	(0.48, 1.03)	0.07
Family history of premature CHD	0.77	(0.67, 0.88)	<0.001
Current smoker	0.99	(0.86, 1.13)	0.91
Diabetes	1.69	(1.47, 1.94)	<0.001
Month (January)	1.00	-	
February	1.02	(0.77, 1.34)	0.88
March	1.15	(0.88, 1.50)	0.28
April	1.20	(0.93, 1.57)	0.15
May	0.94	(0.72, 1.23)	0.67
June	1.07	(0.82, 1.40)	0.58
July	0.95	(0.72, 1.24)	0.72
August	1.04	(0.80, 1.37)	0.73
September	0.94	(0.72, 1.23)	0.68
October	1.03	(0.80, 1.34)	0.76

Jayawardana S, *et al. BMJ Qual Saf* 2020; 29:541–549. doi: 10.1136/bmjqs-2019-010067

November	1.08	(0.84, 1.40)	0.51
December	1.06	(0.82, 1.37)	0.62
Year (2007)	1.00	-	
2008	0.91	(0.66, 1.26)	0.58
2009	1.10	(0.81, 1.50)	0.51
2010	1.10	(0.81, 1.51)	0.51
2011	0.93	(0.72, 1.34)	0.94
2012	1.07	(0.79, 1.46)	0.63
Annual hospital PPCI volume	0.99	(0.99, 0.99)	<0.01
Annual hospital PPCI volume squared	1.00	(1.00, 1.00)	<0.01
**Random-effect standard deviation estimate**	Hospital = 0.24		

Jayawardana S, *et al. BMJ Qual Saf* 2020; 29:541–549. doi: 10.1136/bmjqs-2019-010067

Supplementary Table 7 – Hierarchical logistic regression model, in-hospital mortality with adjustment for DTB times

Variable (reference)	In-hospital mortality Odds-ratio	95% confidence interval	Wald p-value
-Intercept-	-	-	<0.001
Off-hours	1.09	(0.95, 1.24)	0.18
AMG score	1.05	(1.05, 1.06)	<0.001
DTB time (per 10 min increase)	1.06	(1.04, 1.07)	<0.001
Female patient	0.93	(0.81, 1.06)	0.31
Deprivation (most deprived)	1.00	-	
2	0.78	(0.59, 1.03)	0.08
3	0.76	(0.57, 1.00)	0.06
4	0.90	(0.68, 1.19)	0.49
5	0.79	(0.60, 1.04)	0.10
6	0.86	(0.65, 1.14)	0.31
7	0.66	(0.49, 0.89)	<0.01
8	0.70	(0.52, 0.94)	0.02
9	0.74	(0.56, 0.99)	0.04
10	0.63	(0.46, 0.87)	<0.01
Previous AMI	0.92	(0.74, 1.14)	0.45
Previous angina	0.98	(0.80, 1.18)	0.83
History of hypertension	0.84	(0.73, 0.97)	0.02
History/present PVD	1.18	(0.86, 1.63)	0.29
History of stroke/CVD	1.07	(0.85, 1.36)	0.52
History of asthma/COPD	0.99	(0.82, 1.20)	0.95
History of CRF	1.23	(0.90, 1.68)	0.18
History/present hypercholesterolemia	0.76	(0.65, 0.89)	<0.001
Previous PCI	0.99	(0.75, 1.30)	0.95
Previous CABG	0.73	(0.47, 1.13)	0.16
Family history of premature CHD	0.74	(0.62, 0.87)	<0.01
Current smoker	1.16	(0.98, 1.36)	0.07
Diabetes	1.64	(1.39, 1.93)	<0.001
Month (January)	1.00	-	
February	0.91	(0.65, 1.27)	0.60
March	1.15	(0.84, 1.57)	0.36
April	1.16	(0.85, 1.59)	0.33
May	0.92	(0.67, 1.26)	0.61
June	1.04	(0.75, 1.43)	0.79
July	0.87	(0.63, 1.20)	0.40
August	1.11	(0.81, 1.53)	0.48

Jayawardana S, *et al. BMJ Qual Saf* 2020; 29:541–549. doi: 10.1136/bmjqs-2019-010067

September	0.95	(0.69, 1.32)	0.80
October	0.97	(0.71, 1.33)	0.89
November	1.00	(0.73, 1.36)	0.99
December	1.00	(0.74, 1.36)	0.97
Year (2007)	1.00	-	
2008	0.86	(0.59, 1.25)	0.44
2009	0.96	(0.66, 1.39)	0.84
2010	1.03	(0.70, 1.51)	0.85
2011	0.83	(0.57, 1.23)	0.37
2012	1.07	(0.73, 1.57)	0.70
Annual hospital PPCI volume	0.99	(0.99, 0.99)	<0.01
Annual hospital PPCI volume squared	1.00	(1.00, 1.00)	<0.01
**Random-effect standard deviation estimate**	Hospital = 0.46		

Jayawardana S, *et al. BMJ Qual Saf* 2020; 29:541–549. doi: 10.1136/bmjqs-2019-010067

Supplementary Table 8 – Adjusted 30-day and in-hospital mortality by time of admission for PPCI (alternative definition of off-hours [1])

	Adjusted OR[a] for off-hours (95% CI)[2]	Adjusted OR[b] for off-hours (95% CI)[3]
30-day mortality	1.12 (1.01 - 1.25; p=0.03)	1.08 (0.96 - 1.20; p=0.16)
In-hospital mortality	1.13(1.00 – 1.29; p=0.04)	1.07(0.94 – 1.22; p=0.29)

p, p-value; OR, odds ratio

[1] Off-hours defined as admissions during weekends and between 7:00 pm – 6:59 am on weekdays.
[2] Adjusted OR[a] – obtained using a hierarchical logistic regression model that adjusted for AMG risk score, sex, Index of Multiple Deprivation score, previous acute myocardial infarction, angina, peripheral vascular disease, cerebrovascular disease(stroke), percutaneous coronary intervention, coronary artery bypass grafting, chronic renal failure, diabetes, smoking status, hypercholesterolemia, hypertension, asthma/chronic obstructive pulmonary disease (COPD), family history of coronary heart disease, annual hospital PPCI volume and month and year of admission. Hospitals included as random intercepts.

[3] Adjusted OR[b] – all variables from OR[a] plus DTB time

Jayawardana S, *et al. BMJ Qual Saf* 2020; 29:541–549. doi: 10.1136/bmjqs-2019-010067

Supplementary Table 9 - Adjusted 30-day and in-hospital mortality by time of admission [1] for PPCI (for analytical cohort including inter-hospital transfers, day-time only centres and PCI cases with DTB times greater than 6 hours)

n=49,604	Adjusted OR[a] for off-hours (95% CI)[2]	Adjusted OR[b] for off-hours (95% CI)[3]
30-day mortality	1.12 (1.01 - 1.24; p=0.02)	1.08 (0.97 - 1.20; p=0.15)
In-hospital mortality	1.16(1.03 – 1.31; p=0.01)	1.10(0.98 – 1.25; p=0.09)

p, p-value; OR, odds ratio

.

[1] Off-hours defined as admissions during weekends and between 6:30 pm – 7:59 am on weekdays.

[2] Adjusted OR[a] – obtained using a hierarchical logistic regression model that adjusted for AMG risk score, sex, Index of Multiple Deprivation score, previous acute myocardial infarction, angina, peripheral vascular disease, cerebrovascular disease(stroke), percutaneous coronary intervention, coronary artery bypass grafting, chronic renal failure, diabetes, smoking status, hypercholesterolemia, hypertension, asthma/chronic obstructive pulmonary disease (COPD), family history of coronary heart disease, annual hospital PPCI volume and month and year of admission. Hospitals included as random intercepts.

[3] Adjusted OR[b] – all variables from OR[a] plus DTB time

Jayawardana S, *et al. BMJ Qual Saf* 2020; 29:541–549. doi: 10.1136/bmjqs-2019-010067

Appendix 2: Door-to-balloon times and mortality

Patients in longer DTB time categories had higher unadjusted 30-day and in-hospital mortality, ranging from 2.77% 30-day mortality for PPCIs with DTB times within 30 minutes to 7.04% for patients with DTB times between 121-150 minutes (Supplementary Figure 2).

After adjustment for patient risk factors, seasonality and time trend, as well as annual hospital PPCI volume, longer DTB time continued to be associated with a higher likelihood of mortality (Supplementary Figure 3). Estimates from the hierarchical logistic regression model indicated that a 10 minute increment in DTB time increased the odds of 30-day mortality by 4.2% (p<.001). DTB time was also associated with higher in-hospital mortality, where a 10 minutes increase in DTB time increased the odds of in-hospital mortality by 6.2% (p<.001).

Supplementary Figure 2: Unadjusted 30-day and in-hospital mortality rates by DTB time category

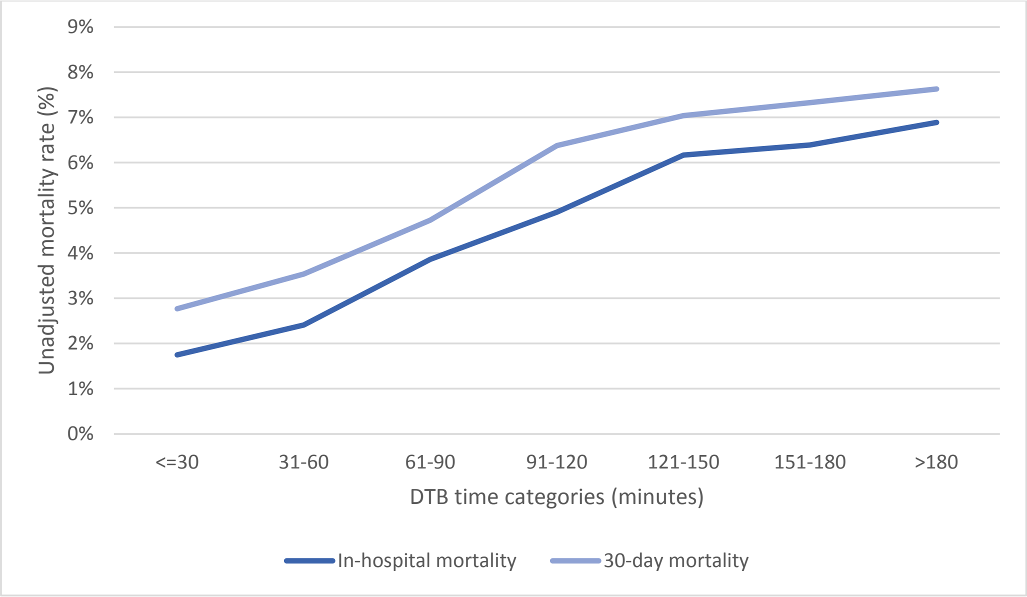

Jayawardana S, *et al. BMJ Qual Saf* 2020; 29:541–549. doi: 10.1136/bmjqs-2019-010067

Supplementary Figure 3: Predicted 30-day and in-hospital mortality from the hierarchical logistic regression model over a range of door-to-balloon times

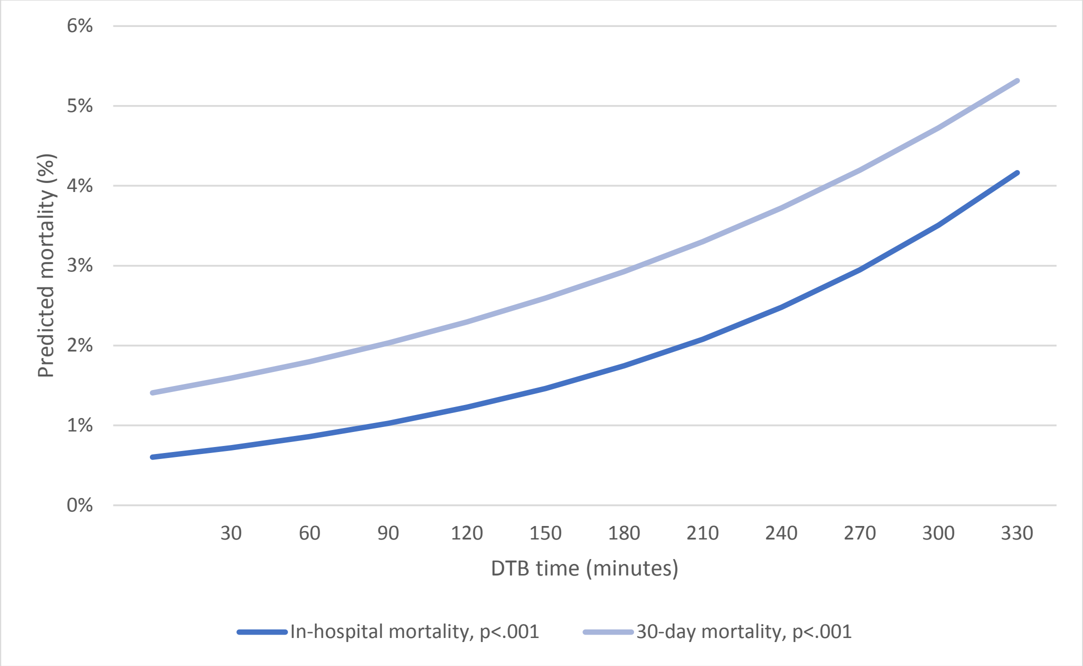

All other covariates held constant.

Jayawardana S, *et al. BMJ Qual Saf* 2020; 29:541–549. doi: 10.1136/bmjqs-2019-010067

References

1 Herrett E, Smeeth L, Walker L, *et al.* The Myocardial Ischaemia National Audit Project (MINAP). *Heart* 2010;**96**:1264–7. doi:10.1136/hrt.2009.192328

2 NICOR. Myocardial Ischaemia National Audit Project (MINAP) Annual Public Report (2012/13). National Institute for Cardiovascular Outcomes Research 2013. https://www.nicor.org.uk/category/minap/ (accessed 15 Dec 2018).
